# Stereotactic Body Radiotherapy for Refractory Ventricular Tachycardia: From Serendipity to Precision Biological Ablation

**DOI:** 10.31083/RCM49589

**Published:** 2026-07-22

**Authors:** Zhoushan Gu, Zhou Xu, Youmei Shen, Linsheng Shi, Hailei Liu, Minglong Chen

**Affiliations:** ^1^Department of Cardiology, Affiliated Hospital of Nantong University, 226001 Nantong, Jiangsu, China; ^2^Department of Cardiology, The First Affiliated Hospital with Nanjing Medical University, 210029 Nanjing, Jiangsu, China

**Keywords:** stereotactic body radiotherapy, ventricular tachycardia, cardiac radioablation, arrhythmia substrate, myocardial fibrosis, electrophysiological remodeling

## Abstract

Ventricular arrhythmias (VAs), particularly refractory ventricular tachycardia (VT), pose a significant therapeutic challenge and are associated with high morbidity and mortality. For patients who do not respond to conventional therapies, including antiarrhythmic drugs and catheter ablation (CA), stereotactic body radiotherapy (SBRT)—also known as stereotactic arrhythmia radioablation (STAR)—has emerged as a promising non-invasive alternative. This review synthesizes current clinical evidence from prospective trials, retrospective series, and case reports that collectively demonstrate the efficacy of STAR in reducing VT burden and improving quality of life. This review also explores the evolving understanding of the associated underlying biological mechanisms, which appear to involve a complex interplay between early electrophysiological conduction reprogramming and delayed structural myocardial remodeling mediated by inflammation and fibrosis. Finally, we address the critical technical considerations, including patient selection, target delineation using multimodal imaging and electroanatomic mapping, motion management, and treatment planning, which are essential for the safe and effective delivery of STAR. While short-term outcomes are encouraging, ongoing research is needed to optimize dosing, refine techniques, and establish long-term efficacy and safety.

## 1. Introduction

Ventricular tachycardia (VT) is a life-threatening cardiac arrhythmia that significantly contributes to global cardiovascular mortality. Despite optimized medication or catheter ablation (CA), more than 50% of patients suffered from death or recurrent VT during a median follow-up of 4.3 years [1]. With continued technological advancements, VTs have become increasingly amenable to multimodal management strategies, including implantable cardioverter-defibrillators (ICDs), antiarrhythmic drugs (AADs), and CA. However, a substantial subset of patients developed therapy-refractory VT, often characterized by incessant episodes or electrical storm despite optimized AADs and CA [2,3]. In these challenging cases, CA can be limited by the deep intramural location of the arrhythmogenic substrate, the presence of extensive epicardial scar, or comorbidities that preclude invasive procedures. Consequently, affected patients frequently experience recurrent ICD shocks, marked deterioration in quality of life, and a significantly increased risk of mortality [4].

In this context, stereotactic body radiotherapy (SBRT) has been introduced as a novel, non-invasive therapeutic strategy. First reported in a landmark case by Cuculich et al. in 2017 [5], SBRT—termed stereotactic arrhythmia radioablation (STAR)—delivers a high ablative dose of radiation precisely to the VT substrate identified through non-invasive or invasive mapping. This approach leverages the well-established principles of SBRT used in oncology but applies them to a functional cardiac target [6]. Over the past seven years, a growing body of evidence from single-center experiences, prospective trials like ENCORE-VT [7] and STARNL-1 [8], and multi-center initiatives such as RAVENTA [9] and STOPSTORM.eu [10] has begun to define its role in clinical practice. This review aims to comprehensively summarize the clinical evidence of STAR for VAs and to elucidate the current understanding of its multifaceted antiarrhythmic mechanisms.

## 2. Clinical Evidence for STAR in VT Patients

### 2.1 Efficacy of STAR for VT Control

The initial proof-of-concept was provided by Cuculich et al. [5], who treated five patients with refractory VT using a single 25 Gy fraction, resulting in a dramatic reduction in VT episodes from a median of 119/month to 0/month at 3 months. This study laid the foundation for subsequent investigations that further validated its efficacy. In the Phase I/II ENCORE-VT trial (N = 19), the efficacy of STAR was further demonstrated. The study reported that VT episodes or premature ventricular contraction (PVC) burden were reduced in 17/18 evaluable patients (94%). The frequency of VT episodes or PVC burden was reduced by 75% in 89% of patients. In addition, an overall survival rate of 89% was observed at 6 months [7]. Similarly, the prospective Dutch STARNL-1 trial (N = 20) demonstrated 87% reduction of treated VT-episodes [8]. These findings have been consistently replicated across multiple case series from diverse regions and centers [11,12,13]. In a recent pooled analysis of 23 studies, treatment with STAR was associated with a greater than 90% reduction in VT episodes and ICD therapies among patients with refractory VTs [14].

Across a broad range of underlying etiologies, STAR appears to be consistently effective in reducing VT burden. Significant reductions in VT episodes have been reported in diverse patient populations, including those with ischemic cardiomyopathy [15], non-ischemic cardiomyopathy [16], Chagas disease [17], and even idiopathic VT [18]. Importantly, therapeutic benefit has also been observed in particularly high-risk clinical scenarios, such as patients presenting with electrical storm or those supported with left ventricular assist devices, in whom STAR was associated with meaningful reductions in VT episodes [19,20,21,22]. Furthermore, in patients experiencing VT recurrence after an initial STAR procedure, successful re-treatment with STAR has been reported, underscoring its potential role as a repeatable therapeutic strategy [23,24].

### 2.2 Effect of STAR on Mortality

The effect of STAR on mortality is much less pronounced than that of reduction in VT episodes. A pooled analysis by Benali et al. [25] reported a one-year all-cause mortality of 32%, with most (52%) deaths attributed to progressive heart failure rather than procedural complications or arrhythmia recurrence. A recent pooled analysis reported combined overall survival rates of 86%, 72%, and 57% at 3, 12, and 24 months, respectively [14]. In contrast, in the VANISH trial [26], which compared catheter ablation with escalation of antiarrhythmic drug therapy in patients experiencing VT despite antiarrhythmic treatment, 1-year mortality was similar between the two groups, ranging from approximately 10% to 15%. It is important to note that patients enrolled in STAR studies generally represent a population with more advanced cardiac disease and higher baseline risk than those included in conventional ablation trials. Therefore, although STAR appears to confer meaningful short-term reductions in VT burden, whether these benefits translate into a long-term mortality reduction remains uncertain.

Overall, the available evidence suggests promising short-term reductions in VT burden; however, the long-term efficacy and safety of STAR remain to be established. Most currently available studies evaluating STAR for VT are small single-center case series or early-phase studies, often with limited sample sizes and relatively short follow-up durations (typically ≤12 months). In addition, there is considerable heterogeneity across studies in terms of patient populations, radiation dose protocols, target delineation strategies, and follow-up periods (Table 1, Ref. [5,7,8,9,10,12,15,19,27,28,29,30]). Such heterogeneity raises concerns regarding potential biases and limits the generalizability of the findings. Therefore, large randomized clinical trials with longer follow-up are needed to further validate the therapeutic role of STAR in patients with refractory VT.

**Table 1.** **A summary of clinical studies evaluating the efficacy of STAR in VT patients**.

Year	Study design	Sample size	Cardiomyopathy	Radiation dose	Key results	Follow-up	References
2017	Single-arm, prospective study	5	ICM: 2 NICM: 3	25 Gy × 1 fraction	99.9% reduction in VT burden; 2 recurrences at 12 months	12 months	[5]
2019	Prospective phase I/II trial	19	ICM: 11 NICM: 8	25 Gy × 1 fraction	VT episodes or PVC burden was reduced by 75% in 89% of patients	12 months	[7]
2021	Single-arm, retrospective study	8	ICM: 4 NICM: 4	15–25 Gy × 1 fraction	ICD therapies decreased from median 69.5 to 13.3	7.8 months	[27]
2020	Single-arm, prospective study	5	ICM: 4 NICM: 1	25 Gy × 1 fraction	All patients experienced recurrence, 2/5 died of heart failure	12 months	[12]
2021	Single-arm, prospective study	8	ICM: 4 NICM:4	25 Gy × 1 fraction	VT events decreased from 29 ± 33 to 11 ± 9 (at 3 months) and 2 ± 2 (at 6 months)	8 months	[15]
2022	Single-arm, retrospective study	17	ICM: 10 NICM:7	25 Gy × 1 fraction	91% reduction in VT burden	12.5 months	[19]
2023	Single-arm, prospective study	7	NA	25 Gy × 1 fraction	5/7 demonstrated a reduction in ventricular tachycardia events	6 months	[28]
2023	Single-arm, prospective study	5	ICM: 2 NICM: 3	25 Gy × 1 fraction	3/5 had a decrease in VT episodes; 1 died of cardiogenic shock	6 months	[9]
2023	Single-arm, prospective study	6	ICM: 6 NICM: 0	25 Gy × 1 fraction	≥50% reduction in treated VT episodes in 4/6 (67%) patients	12 months	[8]
2025	Single-arm, prospective study	3	ICM: 1 NICM: 2	25 Gy × 1 fraction	No VT events were recorded in two patients; one died from progressive heart failure	12 months	[10]
2025	Single-arm, retrospective study	12	ICM: 9 NICM: 3	25 Gy × 1 fraction	64.5% reduction in VT burden; 6/9 recurrences at 6 months	12 months	[29]
2026	Single-arm, prospective study	19	ICM: 8 NICM: 11	25 Gy × 1 fraction	81% reduction in ICD therapies	14 months	[30]

ICD, implantable cardioverter defibrillator; ICM, ischemic cardiomyopathy; NICM, non-ischemic cardiomyopathy; PVC, premature ventricular contraction; STAR, stereotactic arrhythmia radioablation; VT, ventricular tachycardia; NA, not available.

### 2.3 Safety Profile

Based on current studies, the acute toxicity of STAR is generally low [31]. However, because ionizing radiation preferentially injures actively dividing epithelial cells, concerns remain regarding potential late toxicities, particularly given the close proximity of critical thoracic structures. One important concern is the impact of STAR on the heart itself. According to a recent meta-analysis [14], STAR does not appear to significantly impair left ventricular ejection fraction. Nevertheless, individual studies have reported heart failure exacerbation [9,32,33], myocardial injury [8], worsening mitral regurgitation [9], and, rarely, intracardiac thrombus formation [8]. Long-term data on cardiac-specific toxicities—such as radiation-induced coronary artery disease or progressive valvular dysfunction—remain limited and are a focus of ongoing surveillance [34].

Extracardiac complications primarily involve injury to adjacent structures, including the pericardium, gastrointestinal tract, and lungs. The most commonly reported adverse events are pericarditis, pneumonitis, and pleural effusions [14]. These events are generally asymptomatic and transient, rarely requiring additional treatment [14]. In some cases, localized lung fibrosis has been observed, typically limited in extent [35] and clinically silent [15]. Rare but more severe complications—such as gastropericardial fistula [7], esophago-pericardial fistula [35], or phrenic nerve palsy [36]—have also been reported and warrant particular attention.

Because the vast majority of patients undergoing STAR have implanted cardiac devices, the interaction between radiation therapy and device function has been carefully evaluated. Overall, STAR appears to be safe in patients with implanted devices. However, rare instances of device reset have been reported [8], underscoring the need for careful peri-procedural device monitoring.

Based on currently available studies, acute toxicity associated with STAR appears to be relatively low; however, long-term safety remains an important concern. Given the relatively short follow-up periods in most published studies, reports of late complications remain limited. Nevertheless, several potential late adverse events have been described, including aortic valve disease (stenosis), radiation-induced coronary artery disease, conduction system injury, delayed cardiomyopathy, pericardial disease, bronchopulmonary hemorrhage and chest wall pain [33]. As many patients may survive for several years following treatment, the long-term cardiac effects of radiation remain an important and unresolved issue that warrants continued investigation.

## 3. Underlying Antiarrhythmic Mechanisms

In oncology, SBRT achieves tumor control mainly by inducing lethal genomic injury and secondary microvascular and immunologic effects. However, cardiomyocytes are terminally differentiated cells with condensed chromatin and no ongoing deoxyribonucleic acid (DNA) replication, making them intrinsically resistant to radiation-induced DNA damage. As a result, the therapeutic effects of STAR in arrhythmia management are mediated by mechanisms that are distinct from those responsible for tumor eradication. The precise mechanisms by which STAR terminates VT are complex and appear to operate on two distinct temporal scales: an early functional effect and a late structural effect.

### 3.1 Early Electrophysiological Reprogramming

Surprisingly, clinical antiarrhythmic effects are often observed within days of treatment, preceding any significant tissue fibrosis [37]. This phenomenon facilitates the assessment of the acute electrophysiological effects of STAR. Preclinical and translational studies have shed light on this phenomenon. Zhang et al. [38] demonstrated in a porcine model that a single 25 Gy dose of radiation leads to an upregulation of key ion channels (Nav1.5, Cav1.2) and gap junction proteins (Cx43) within 24 hours, probably via Notch signaling, effectively affecting action potential and conduction velocity without causing transmural fibrosis at this early stage. This “electrical conduction reprogramming” is believed to disrupt the re-entrant circuits responsible for VT [39]. Recent work by Mages et al. [40] provides direct human evidence that STAR acutely affects cardiomyocyte electrophysiology, STAR modulates the expression of various ion channels, calcium handling proteins, and gap junction proteins. Thus, existing evidence suggests that, in the acute phase, STAR may modulate cardiac electrophysiology through effects on ion channels and gap junction.

Although electrophysiological reprogramming has been proposed as a potential mechanism underlying the early antiarrhythmic effects of STAR, the current mechanistic understanding remains incomplete. Evidence supporting rapid ion channel remodeling in humans is still limited. Alternative mechanisms have been proposed to explain the early reduction in VT burden observed after STAR. These may include modulation of cardiac autonomic activity, radiation-induced inflammatory responses, and transient conduction block within arrhythmogenic substrates [41]. Therefore, the relative contribution of these mechanisms remains uncertain, and further mechanistic studies are required.

### 3.2 Structural and Neural Remodeling

Over weeks to months, the primary mechanism shifts towards structural remodeling. Histopathological examination of human myocardium post-mortem revealed a biphasic response: an initial inflammatory phase dominated by macrophage infiltration, followed by a proliferative phase characterized by dense collagen deposition and fibrosis [42,43]. This process effectively creates a non-conductive scar that permanently isolates or destroys the arrhythmogenic substrate, analogous to the goal of CA but achieved through a different biological pathway [44]. The extent of this fibrotic remodeling appears to be dose-dependent, as suggested by early human imaging studies correlating radiation dose with late gadolinium enhancement on cardiac magnetic resonance imaging (MRI) [45].

Additionally, STAR has been reported to exert neuromodulatory effects. Emerging evidence suggests that radiation may influence cardiac autonomic nerves and neural signaling [46]; however, the extent and durability of these effects, as well as their contribution to arrhythmia suppression, remain incompletely understood.

## 4. Critical Technical Considerations for STAR Delivery

Although STAR is considered a non-invasive approach, its potential effects on surrounding tissues necessitate precise and carefully controlled application. The successful and safe application of STAR hinges on a multidisciplinary workflow that addresses several unique challenges.

### 4.1 Patient Selection

Current safety and efficacy data for STAR in VT control are largely derived from patients with refractory VT who are either not eligible for catheter ablation or have failed catheter ablation and/or AAD therapy. Therefore, based on the available evidence, careful patient selection is paramount and should prioritize individuals with truly refractory VT and a reasonable life expectancy [47].

### 4.2 Target Delineation

Accurate target volume definition is the cornerstone of STAR. The gross tumor volume is replaced by the “gross arrhythmia volume” (GAV), which is delineated with a combination of both imaging and mapping data: (1) Electroanatomic Mapping (EAM): Both endocardial and epicardial voltage maps from prior CA procedures are accurate approaches for defining scar (typically <1.5 mV) [48,49]. (2) Non-invasive Imaging: Cardiac magnetic resonance with late gadolinium enhancement (LGE-CMR) is invaluable for visualizing myocardial scar, especially in non-ischemic substrates [50]. Nuclear imaging can identify areas of metabolic mismatch or sympathetic innervation [51]. (3) Electrocardiogram (ECG) Imaging: Non-invasive electrocardiographic imaging (ECGI) can localize the origin of VT [5,52]. Consensus statements provide detailed guidance on integrating these data sources into a unified GAV [47,53]. In brief, the GAV should cover the VT exit site and colocalized ventricular scar [8].

### 4.3 Treatment Planning and Dose Constraints

A typical prescription is a single fraction of 25 Gy (varied in different studies) to the planning target volume (PTV), which is the GAV plus a margin to account for setup uncertainties and residual motion [54]. However, dose escalation is being explored in trials like DEFT-STAR (NCT05594368) to potentially improve efficacy [55]. Stringent dose constraints for organs-at-risk (OARs) are critical to avoid toxicity. Key constraints include limits for the coronary arteries (<12–15 Gy to a small volume), esophagus, lungs, and spinal cord, often adapted from thoracic SBRT guidelines [56,57]. 25 Gy is used currently to irradiate targeted regions of the left ventricle only, whereas lower doses, e.g., 5 Gy have been used for whole-heart irradiation in limited pre-clinical and clinical studies [58]. Multi-platform benchmarking studies, such as those from the RAVENTA consortium, are working to harmonize these planning practices [59].

### 4.4 Treatment Delivery and Motion Management

After all radiotherapy plans had passed routine physics quality assurance based on calibrated phantoms, patients were immobilized. Stereotactic radiotherapy ablation was then delivered using a linear accelerator equipped with an image-guided radiotherapy system, with real-time beam alignment achieved through cone-beam computed tomography to ensure precise registration with the target volume.

Cardiac and respiratory motion present a major challenge for precise targeting. Several strategies have been developed: (1) Mid-Position Planning: This technique uses a time-weighted average of the cardiac cycle to create a more stable target, reducing the need for large internal margins [60]. (2) Gating: Real-time ECG gating can deliver radiation only during a specific phase of the cardiac cycle (e.g., diastole), minimizing motion [61]. (3) Tracking: Robotic platforms like CyberKnife use real-time tracking of fiducial markers or the target itself (e.g., via Synchrony® system) to dynamically adjust the beam, allowing for sub-millimeter accuracy even with continuous motion [62,63].

## 5. Knowledge Gaps and Future Directions

Ever since the first report by Cuculich et al. [5], an increasing number of clinical and preclinical studies have demonstrated the feasibility, efficacy, and short-term safety profile of STAR, and have begun to explore the mechanisms underlying its antiarrhythmic effects. However, as the existing evidence is largely derived from single-arm studies or small case series, several critical questions remain unresolved. These include: (1) efficacy and safety—whether STAR provides incremental or superior benefit compared with conventional therapies across diverse patient populations; (2) treatment optimization—the optimal radiation dose, fractionation, and workflow required to maximize efficacy while ensuring long-term safety; and (3) mechanistic understanding—the detailed biological and electrophysiological mechanisms responsible for the antiarrhythmic effects of STAR.

With these gaps in mind, future research should focus on several key areas: (1) the establishment of international registries and well-designed clinical trials to generate robust long-term safety and efficacy data; (2) the development of reliable biomarkers to predict treatment response and guide patient selection; (3) prospective optimization of radiation dose and fractionation schemes; (4) further refinement of motion management and image-guidance technologies to enhance targeting precision; and (5) basic and translational studies to elucidate the short- and long-term mechanisms of STAR-mediated arrhythmia suppression. The ultimate goal is to integrate STAR into a standardized, multidisciplinary care pathway for patients with refractory ventricular arrhythmias.

## 6. Conclusion

Stereotactic arrhythmia radioablation represents a paradigm shift in the management of refractory ventricular tachycardia. A growing body of clinical evidence supports its efficacy in significantly reducing arrhythmia burden and improving quality of life in a highly selected, otherwise untreatable patient population. Its antiarrhythmic action is now understood to be dual-phasic, involving both an early, functional disruption of electrical conduction and a late, structural creation of a fibrotic scar. The safe and effective delivery of STAR requires a meticulous, multidisciplinary approach to patient selection, target delineation, motion management, and treatment planning. While long-term data with larger sample size are still maturing, STAR stands as a powerful addition to the electrophysiologist’s armamentarium against this formidable clinical challenge.
